# A Wider Field

**Published:** 1920-06-26

**Authors:** 


### A WIDER FIELD

It is not necessary here to press the urgency of the appeal which this number of THE HOSPITAL will carry to all parts of the country, for that is done both directly and indirectly in other sections of the journal. Also there has been within the last few months no scarcity of illustration that the present position of the voluntary hospitals is one causing unexampled anxiety. Their position may not everywhere be desperate, but the financial outlook is decidedly serious.

There is, however, one sign to which we would draw attention, and one which portends a development which we feel prompted to urge by every means in our power. Not only is it becoming increasingly realised by the masses of the country that in their hour of need any one of them may want the help of the best that medical or surgical skill can give, but also that to ensure the availability of such assistance it is vitally to their own interest that its source and the whole system of its development should receive their willing and sympathetic support. Among all classes of society some degree of awakening is now apparent, and although, just as it has crippled the hospitals themselves, the war has seriously handicapped many of those people who used to help them freely, yet it has brought both realisation and opportunity to a vast number of our citizens, who with but relatively little organised guidance can, in part at least, make good the deficiency.

To detail the social classes and the various ways by which we may gain their sympathies is beyond the scope of this note. Week by week our pages touch upon one or other aspect of the problem. But to the great middle classes, whose members so often complain that the labourer can now afford and can now well pay for the medical benefits he may in emergency require, let us point out that, in common with many similar statements of the day, this represents by no means the whole truth. It is not a question of the working man being the chief delinquent. We are a long way off eliminating poverty from our midst. We are still far, as a reliable contemporary says, "from possessing a working class able fully to pay for such aid as the hospitals can give, even though workers may be more prosperous than their forefathers were in the days when the hospitals were founded." That they are beginning to pay something we know; in several quarters graduated payment is being quite justifiably instituted. But the hospital classes cannot alone be expected to pay all that would make up the deficit, and we cannot in justice refrain from deprecating the accusation of their indifference. The situation as it really is demands a wholehearted effort on the part of *all* members of *all* classes. An effort of determination to give, through one of the channels described in these pages to-day, something—(something in accordance with their individual means is all we ask)—to the cause of the voluntary hospitals. And this must be done not simply as a single gift to meet some part, great or small, of the crisis of the moment, but must be considered as an annual duty and a just annual contribution to the support of the medical service, which all in their hour of sickness may require.

Direct matters of public health require, above all things, public enlightenment and education; so also these indirect matters of institutional support. In past years no journal has done more than THE HOSPITAL to disseminate knowledge and appreciation of institutional matters in those quarters where it was likely to produce the richest harvest in voluntary support. Since the war the fields of possible fertility have enormously widened. They include the whole country; and in future, as always, it will be our earnest endeavour still to fulfil our task and our policy of public education, and to deal with all questions involved in the maintenance of an adequate hospital system. This week THE HOSPITAL will come to the hands of many who will find expressed in this journal, week by week, on vital health questions of the moment, expert opinions which have no special politics to serve and no other aim than the general well-being of the public.

